# Emerging technologies to measure neighborhood conditions in public health: implications for interventions and next steps

**DOI:** 10.1186/s12942-016-0050-z

**Published:** 2016-06-23

**Authors:** M. Schootman, E. J. Nelson, K. Werner, E. Shacham, M. Elliott, K. Ratnapradipa, M. Lian, A. McVay

**Affiliations:** Department of Epidemiology, College for Public Health and Social Justice, Saint Louis University, 3545 Lafayette Avenue, Saint Louis, MO 63104 USA; George W. Brown School of Social Work, Washington University in St. Louis, Saint Louis, MO USA; Department of Behavioral and Science and Health Education, College for Public Health and Social Justice, Saint Louis University, Saint Louis, MO USA; Department of Biostatistics, College for Public Health and Social Justice, Saint Louis University, Saint Louis, MO USA; Division of General Medical Sciences, Department of Medicine, Washington University School of Medicine, Saint Louis, MO USA

**Keywords:** Neighborhood, Geographic locations, Residence characteristics, Intervention studies, Public health informatics, Environment and public health, Social media

## Abstract

Adverse neighborhood conditions play an important role beyond individual characteristics. There is increasing interest in identifying specific characteristics of the social and built environments adversely affecting health outcomes. Most research has assessed aspects of such exposures via self-reported instruments or census data. Potential threats in the local environment may be subject to short-term changes that can only be measured with more nimble technology. The advent of new technologies may offer new opportunities to obtain geospatial data about neighborhoods that may circumvent the limitations of traditional data sources. This overview describes the utility, validity and reliability of selected emerging technologies to measure neighborhood conditions for public health applications. It also describes next steps for future research and opportunities for interventions. The paper presents an overview of the literature on measurement of the built and social environment in public health (Google Street View, webcams, crowdsourcing, remote sensing, social media, unmanned aerial vehicles, and lifespace) and location-based interventions. Emerging technologies such as Google Street View, social media, drones, webcams, and crowdsourcing may serve as effective and inexpensive tools to measure the ever-changing environment. Georeferenced social media responses may help identify where to target intervention activities, but also to passively evaluate their effectiveness. Future studies should measure exposure across key time points during the life-course as part of the exposome paradigm and integrate various types of data sources to measure environmental contexts. By harnessing these technologies, public health research can not only monitor populations and the environment, but intervene using novel strategies to improve the public health.

## Background

Adverse neighborhood conditions affect various health outcomes and play an important role beyond characteristics at the individual level [1]. Characteristics of neighborhood conditions can be classified in multiple aspects, including the built/physical environment (e.g., availability of sidewalks), social and economic conditions (e.g., poverty rate), availability of medical care (e.g., primary care physicians per population), and environmental determinants (e.g., organic and inorganic pollutants) [2]. There is increasing interest in identifying specific characteristics of the social and built environments adversely affecting health outcomes. This is exemplified by recent initiatives, such as personalized or precision medicine [3]. The Precision Medicine Initiative is a comprehensive effort to better understand which treatments work for which individuals and under which conditions [4]. By also harnessing environmental exposures, a much more comprehensive view of the population can be developed because the collective health is shaped by factors beyond clinical care and/or genetic predisposition [5]. Some called the community-based corollary “precision public health” [6]. “Achieving health equity” and “creating social and physical environments that promote good health for all” are two of the four Healthy People 2020 goals that provide the impetus to examine geographically based disparities related to adverse neighborhood conditions.

Because environmental exposures are responsible for the majority of risk for many diseases, systematic and comprehensive measures of these exposures need to be obtained. Most research has assessed aspects of the built and social environmental exposures via self-reported instruments or used census data. In contrast to self-reported measures, field audits involve observers documenting specific characteristics of the built environment with checklists and require a visit to each area of interest. These are expensive and time-consuming options, especially for geographically dispersed places, hindering the generalizability of studies beyond relatively small geographic areas. Administrative sources (e.g., census data) often are based on arbitrary boundaries that may not represent neighborhoods. In contrast to relatively stable forms of neighborhood conditions (poverty, racial segregation, etc.) that are typically measured using these approaches, potential threats in the local environment such as crime and the response to crime may be subject to short-term change that can only be measured with more nimble technology.

The advent of newly developing technologies such as webcams, social media, or crowdsourcing may offer new opportunities to obtain geo-spatial data about neighborhoods that may circumvent the limitations of traditional data sources used in neighborhood research. New methods and technologies to measure exposures should be developed that provide a more balanced approach to the measurement of the gene-environment equation [7, 8]. The imbalance in precise measurements of genes and variable measurement of environments may result in an inability to fully derive the public health benefits from expenditures on the human genome. This paper describes the utility, validity and reliability of selected emerging technologies to measure neighborhood conditions for public health applications. It also describes next steps for future research and opportunities for interventions.

### Google Street View

Omnidirectional imagery measures aspects of the built and social environment and refers to simultaneous collection of images in multiple directions from a single location, producing a panoramic view (e.g., Google Street View). It provides a visual record of an area and allows observation of neighborhood characteristics. Google Street View has generally been found to be reliable and valid relative to the gold standard of in-person audits in urban areas [9–15]. Moreover, neighborhood conditions measured using Google Street View are associated with observed physical activity and children’s antisocial behavior [16, 17].

Advantages of Google Street View include efficiency, researcher safety, low cost, unobtrusive data collection and access to historical images of locations. Google Street View and similarly collected data offer an efficient alternative, particularly if audits are needed over large or geographically dispersed areas. The ability to “go back in time” is an important advantage for longitudinal research that cannot be implemented through in-person approaches. Google Street View offers the potential for longitudinal comparison at a finer spatial scale and from a ground-level perspective that may capture features not visible from aerial and satellite platforms.

Limitations of Google Street View also exist. First, Google Street View focuses on the built environment and does not provide data about specific environmental contaminations. Second, the level of agreement between virtual and field audit is in general lower for the assessment of subjective than objective dimensions of the environment, including environmental aesthetics (e.g., presence of garbage, graffiti) and condition of the sidewalks (e.g., sidewalk width, alignment) [9, 11, 18]. Third, visual interpretation is limited to the camera’s perspective and vantage point, so auditors may not be able to view features as closely as they would in the field. Fourth, images provided by Google Street View are collected at a particular point in time, limiting its utility when imagery is not available for a time period of interest. Google’s release of historical Street View imagery in the spring of 2014 offers new opportunities for understanding temporal change at locations where historical imagery exists. Some measures (e.g., the presence of litter) may be affected by time of day, which may or may not be observed by Google Street View data. Also, Google Street View may not be available at all locations; up to 40 % of segments selected in a metropolitan sample were not available [19]. This limitation will become less of a problem as Google continues to expand the geographic coverage to more streets. Studies have also identified some imagery date disruptions in Google Street View data that may be problematic for virtual audits or spatial processing of these data for environment-behavior research [20]. Finally, the validity and reliability of Google Street View in rural areas is limited [13].

### Webcams

Web cameras (webcams) are video cameras that capture and stream images in real time to a computer or computer network. Webcams provide the ability to conduct large scale surveillance of multiple geographic locations with minimal personnel burden. Currently, the largest and most accessible collection of environmental webcam image data is the Archive of Many Outdoor Scenes (AMOS; http://amosweb.cse.wustl.edu/). Established in 2006, AMOS is a webcam resource providing open access to over 29,000 webcams across the world with 847 million images and counting [21, 22]. AMOS captures images of outdoor spaces and allows for investigation of changes in built environment over time as well as examination of how individuals interact within the environment. Webcams have been shown to be a reliable and valid measure of built environment characteristics [23]. Currently, public health research utilizing webcams to assess the built environment is sparse but burgeoning. Webcams have been demonstrated as successful tools to monitor use of active transportation—alking and biking—which are important health-promoting physical activities [21, 24].

Webcams provide a number of advantages including continuous monitoring of the built environment that is relatively low in cost and minimally obtrusive. Continuous monitoring of different locations allows for both longitudinal and cross-sectional research investigating how the built environment can impact the population over time or how different environments compare without introducing the artifact of observer or participant bias that is often a limitation of in-person audits. Furthermore, the use of existing infrastructure is valuable and it comes at little to no cost for the researchers.

However webcams are not without limitations. When utilizing images from existing webcams, inconsistencies in image properties (i.e. sizes, refresh rates, scenes) are of concern and the data available is limited to those locations with existing cameras. Factors such as weather, obstructed views, and mechanical malfunction can limit the reliability of the images as well. Additionally, webcams produce a large number of images and data to process and classify. However, this limitation can be overcome by using computer-based algorithms to detect anomalies of interest for evaluation [25] or by coupling webcam technology with crowdsourcing initiatives to quickly provide researchers with high quality and inexpensive data [24].

### Crowdsourcing

Another innovative and efficient manner for harvesting geo-referenced data is through crowdsourcing or ‘participatory sensing’ applications that rely on citizen participation to achieve their goals. These applications generate real-time updates of the earth as GPS traces are sent via Internet-enabled mobile devices. Crowdsourcing is particularly interesting for neighborhood research as millions of users are contributing content such as photos, videos, notes, and social commentary to their various locations, rendering data that is rich in quantitative and qualitative properties through applications such as Google Earth (www.google.com/earth/) and OpenStreetMap (www.openstreetmap). For example, these data sources have been used to measure motor vehicle traffic updates, audio samples to measure citywide noise pollution [26], and to monitor drug safety surveillance (https://medwatcher.org/), among others. Open source platforms such as GeoChat and Ushahidi, which permit interactive mapping of crowdsourced data (including Web forms, e-mails, short message services [SMS] text messages and Twitter tweets), have gained traction as they can be leveraged to aid in times of natural disaster, disease outbreaks, and to send alerts in real-time [27, 28]. The combination of crowdsourced data and rigorous data analysis via open source geographic information system (GIS) software is appealing, particularly in low resource settings [29].

Advantages of crowdsourcing include real-time data collection, data that is both time stamped and georeferenced, and the accessibility of data for analysis. The ability of crowdsourcing to monitor environments and disease phenomena in real time among large populations is unparalleled. Instant access to location-based social commentary and spatio-temporal movements of phenomena supply rich data that may provide the power to detect associations that cannot be seen with less frequent or lower resolution data sources.

The main limitations of crowdsourced data are the reliance on self-reported data, the need to sift through large amounts of data to obtain appropriate exposure/disease measures, and the potential for selection bias as many people do not actively contribute to crowdsourced data thereby leading to under/over reporting of events. The current emphasis on big data and bioinformatics in public health will hopefully reduce the processing time and interpretation of large quantities of data in the near future [30, 31]. Notably, the wide adoption and usage of web-enabled devices (e.g., smartphones and tablets) among all age groups is likely to continue and thereby increase the representativeness of crowdsourced data [32]. Another limitation is that low-resource settings may have limited access to the necessary mobile cellular networks. However, efforts conducted in parallel research fields (e.g., telemedicine) have successfully used mobile networks in low-income countries such as Nepal, Botswana, and Bolivia to deliver health care and communicate with physicians remotely [33–35]. Thus, the potential of crowdsourcing in similar settings is promising.

### Social media

The use of social media has expanded greatly in the past decade and is producing massive amounts of data. With more than 1 billion users worldwide, Facebook™ is the largest social media network. With the launch of Twitter™ in 2006, daily chatter, conversation, information sharing, and news commentary have become easily accessible. Social media provides user-generated data that can be collected and analyzed by researchers to examine opinions or social norms around specific topics, including health-related foci. Twitter has been used primarily to examine discussions about alcohol and marijuana use [36], e-cigarettes, hookah use, depression [37], and suicide [38–41]. Twitter and other social media outlets have been used to assess the positive and negative nature of posts, specifically for health conditions like depression and schizophrenia [42]. They have also been used to locate where health conditions are most frequently mentioned [43]. Twitter has been demonstrated to be more effective than traditional surveillance methods to identify the 2009 H1N1 pandemic [44].

Some tweets, posts on Twitter, are able to be georeferenced. Twitter data showed that Adderall was tweeted with higher frequency around college campuses relative to outside those areas, particularly in the northeastern United States [45]. Twitter also found that areas designated as food deserts had a lower proportion of tweets about healthy food with a positive sentiment and higher proportion of unhealthy tweets [46]. Exposure to a healthful food environment in an individual’s immediate vicinity facilitates healthful choices while showing that an obesogenic food environment may not necessarily increase the likelihood that individuals will patronize fast food restaurants [47]. Other georeferenced social media, such as Flickr™, have been used to identify characteristics of specific areas using spatial clustering algorithms [48–50]. Few studies have validated neighborhood conditions. Quercia and colleagues showed that smells about industry, transport, and cleaning correlated with air quality indicators using georeferenced picture tags from Flickr and Instagram, and georeferenced tweets from Twitter [51]. Facebook and other social media outlets have been used to recruit research participants in specific areas, which may allow for increased access to georeferenced data and the potential to explore implementation and evaluation of public health interventions [52–55].

Challenges in using social media for public health research related to neighborhood conditions include access to data that is not publicly available and the lack of population-based representation of users. For example, only some public posts on social media are accessible and able to be georeferenced. Despite the large volume of data generated by the many social media users [56], young adults, African Americans, urban/suburban residents, and mobile users use Twitter at higher rates [57]. However, less than five percent of tweets included specific geographic locations [45, 46]. The location information retrieved by built-in GPS receivers might also be inaccurate for various reasons. Furthermore, users can individually choose to add their precise location to a tweet or attach general location information (such as a city or neighborhood). This might result in imprecise and coarse location information of geotagged tweets. Individuals who allow their social media posts to be located are likely different from those who did not, which may lead to selection bias. The distribution of social media use may also be spatiotemporally heterogeneous because users do not contribute equally across space and time. Tweets and other social media use vary across different real-world scale levels (country, city, etc.) and might result in sparse data coverage for some geographical areas [48]. Finally, some users may have multiple accounts and may make comments on neighborhood conditions outside the area they are in. Thus, analyses using social media data should not overstate its representativeness among all residents within a defined geographic study area.

### Unmanned aerial vehicles (drones)

Unmanned aerial vehicles (UAVs), also referred to as drones or as unmanned aerial systems (UASs), have experienced a surge in abilities and availability over the past few years. Drones are starting to be used in public health applications, such as identifying locations for targeted soil sampling to detect chemical spills and areas where the soil is contaminated [58, 59]. Drones have also been used to investigate the causes of infectious disease; explaining a surge in malaria caused by human incursion and carrier species displacement [60], and identifying spatial determinants of tuberculosis in Spain [61]. They have also been fitted with near infrared cameras to detect the biomass of forest areas [62, 63], as well as with lightweight gamma-spectrometers to assess radioactive contamination and the effectiveness of decontamination efforts near the Fukushima Daiichi Nuclear Power Plant in Japan [64].

Drones have several advantages over other methods of observation and/or environmental sampling, and have already been proven to be useful tools in public health. A study using drones to detect copper-contaminated soil found that the benefits of drones included lower flight altitude than normal planes (25 m in their study) allowing for high resolution photos, and faster speed compared to normal planes (4 m/second in their study) [58]. In addition, drones are capable of obtaining high quality image data. The highest spatial resolution from satellite data (commercially available) is 41 cm; however, drones can capture images at a resolution of 4–20 cm [60]. This increased spatial resolution proved very useful in the Fukushima radiation contamination assessment trial, along with the protection of humans from radiation exposure which occurs with manned flight or ground survey work [64]. As drone prices continue to decline and flight performance continues to improve, inexpensive UAVs will become more available for wider use in public health research [65]. Currently, the main attraction of drones is their ability to obtain data in real time and to repeatedly sample study areas as frequently as needed [60].

Drone development is occurring at a rapid pace, and several promising innovations are currently being developed that could prove useful in measuring neighborhood conditions. For example, drones may be used to remotely inspect personal compliance with guidelines (such as wearing personal protective equipment), and measuring stressors such as heat, cold, radiation, pollution, and noise over time [66]. This environmental monitoring ability will be enhanced by solar powered drones with extended run time that are currently under development [67]. Drones have been used to transport automated external defibrillator (AED) devices to people under cardiac arrest. Drone use is planned for rapid inspection of bridges and roads [66], suggesting that neighborhood condition audits could also be accomplished by drones. Some have suggested that drones can locate people and monitor the movement of human populations [68], which suggests that drones could be used to assess the neighborhood social as well as the built environment.

There are several concerns regarding drone use, including safety and privacy. Drones are currently poorly regulated, and as such, pose a risk of varying degree [69]. For example, the misuse of drones concerns include invasion of privacy, either in the pursuit of journalism or of voyeurnalism (for example by paparazzi), or through voyeurism [69]. The number of drone incidents reported to the United States Federal Aviation Administration has increased from 238 total in 2014, to 1133 through November in 2015 [70]. To help alleviate some of these concerns, the United States Federal Government is now requiring the registration of most drones [70]. Additionally, current United States federal regulations limit the autonomous flight of drones, and require that they remain in sight of the operator [70]. Other areas also currently regulate drone usage or are developing regulations including Europe through the European Aviation Safety Agency (EASA), and Australia through the Civil Aviation Safety Authority Regulations (CASR-101) [69]. Additionally, international guidelines are being developed that will help inform regulations regarding drones by the International Civil Aviation Organization (“unmanned aircraft systems [UAS]”, ICAO Circular 328-AN/190, 2011), and by the Joint Authorities for Rulemaking on Unmanned Systems (JARUS Press Release No. 2016/13, April 25, 2016). As rules and regulations regarding the permissible utility of drones are still under debate, the extent of their potential use in public health research remains in question.

### Lifespace measurement

Lifespace refers to the geographic space in which a person lives and functions [71]. Lifespace arose from studies of aging and mobility impairment but has been associated with broader health and functional capacity, including quality of life, resource availability, social interaction, and travel patterns [71]. Traditionally assessed via self-report, mobile technology provides an opportunity to passively measure lifespace with greater precision and accuracy, reducing potential recall and social desirability bias. Using a lifespace approach may help identify the appropriate geographic area/resolution to be used as the basis for deriving exposure measures based on a person’s travel pattern. It may also move toward a people-based rather than a place-based understanding of exposure and context [72]. Proof of principle studies using GPS technology and accelerometer apps in mobile phones established that the technology can reliably capture community-level movements for various populations [73–75]. With the addition of Bluetooth beacons, lifespace measurement can be scaled for room-level, in-home measurements [74].

Strengths of this approach include relatively low cost, low participant burden, objective measurements, and the ability to analyze lifespace as time-series measurements to create a variety of metrics including visual representations. Limitations include potential data loss due to phone battery life (and need for participant to charge the phone each night) and participant decisions about when to turn the device on/off which may result in underreporting of less-active times of day. In addition, the use of mobile phones does not indicate whether the participant needed assistance in travel, which is typically collected using the lifespace self-report measure.

## Future directions and opportunities for interventions

To-date, most studies examining neighborhood characteristics have been limited by: (1) a focus on residence only when most people spend one-third of their time elsewhere; (2) failure to consider cumulative exposures over time (e.g., residential history); and (3) use of arbitrary administrative units (county, census tract, or zip code) to infer neighborhood risks. Future studies should measure exposure across key time points during the life-course as part of the exposome paradigm [2, 8, 76, 77] and integrate various types of data sources to measure environmental and community contexts (such as work, residential, and daily-life settings) in addition to biological and social factors [7]. A GIS is ideally suited to integrate various types of data across multiple levels, recognizing that specific challenges need to be overcome related to ‘big data’ issues particularly when using small geographic areas and a life-course perspective. Merging appropriate methods from various disciplines, including epidemiology, health behavior, genetics and geography will provide a more complete understanding of the spatial interactions that result in spatial patterns of disease. A new paradigm is needed to assess how a lifetime of exposure to environmental- and community-level factors affects the risk of disease [78]. One such paradigm may be eco-geographic genetic epidemiology that integrates various disciplines into models of geographic disease etiology [79].

Combining the digital traces that remain as people interact with the world through smart phones and the aforementioned emerging technology may now provide unprecedented methods to assess a range of environmental factors objectively and with minimal expense and burden to participants. Ecological Momentary Assessment may focus on tracking individuals and events of interest (e.g., smoking, drinking alcohol), but may also collect data about neighborhood conditions through Google Street View and social media (e.g., noise, smells) as persons interact with their environment because smart phones are equipped with GPS technology. However, detailed knowledge about the whereabouts of people and their behaviors as they interact with the world with high spatial and temporal resolution is largely unexplored. The spatiotemporal analysis of Location Based Social Networks has great potential to help better understand the processes of behavior and explore the impact of spatial structures on human activity [80, 81]. Social media posts may also be used to examine the effect of crowd behavioral patterns (e.g., physical activity) and urban characteristics (e.g., built environment) on individual behavior [82].

Real-time interventions use mobile technology to deliver interventions to individuals as they go about their daily lives. These Ecological Momentary Interventions are provided to people during their everyday lives (i.e., in real time) and in natural settings (i.e., real world) [83], are starting to be developed and tailored to individual characteristics, although few are evidence based [84, 85] or location dependent. Perhaps interventions delivered via mobile devices at the time when the user needs it in a high-risk situation (e.g., Flu on Call™) [86] or when struggling to avoid violating a behavior-change goal can be triggered based on the passively sensed geographic location of individuals [87]. For example, when a patient leaves residential treatment for alcohol use disorders and nears a bar, the mobile device initiates an alert asking the patient if s/he wants to be there [88]. A recent study developed a mobile phone application and supporting architecture, in which machine learning models predicted patients’ mood, emotions, cognitive/motivational states, activities, environmental context, and social context based on at least 38 concurrent phone sensor values (e.g., global positioning system, ambient light, recent calls), which showed important effects on major depressive disorder diagnosis [89]. While most research has focused on addiction, future research could examine the effectiveness of interventions focused on other behaviors that also contain a locational component, such as obesity-related disorders (e.g. obesity, diabetes, cardiovascular disease). Mobile phone sensors can be used to develop context-aware systems that automatically detect when patients require assistance. For example, a context-aware system gathered observations about participants and their environment, developed an algorithm to inductively “learn” the relationship between sensor data and the participant’s reported social context, activity, location, and internal states and subsequently intervened based on these predictions [89]. Issues of confidentiality may play a role in this type of research depending on the type of behavior examined. By harnessing real-time data from social media, local programs can be evaluated as they are implemented, generating timely feedback to assess the effectiveness of interventions to improve health outcomes. Such adaptive designs using accumulating data to modify the intervention’s course have been used infrequently in community-based evaluations [90, 91].

Georeferenced social media responses may also help identify where to target intervention activities. For example, Twitter analysis may drive an intervention focused on reducing Adderall use around college campuses [45] or georeferenced tweets could focus on healthy food options in food-desert-identified areas [46]. Tweets may be used to evaluate changes before and after implementation of local interventions, recognizing that only a select minority of individuals in specific areas allow their tweets to be georeferenced.

Other emerging technologies may also be used to passively evaluate the effectiveness of interventions. For example, webcams may be applied to public health intervention research by monitoring individual- and community-level interaction with the built environment. Additional applications could include the use of webcams to monitor differences in the use of green space and outdoor activity based on neighborhood characteristics and how changes to the built environment can impact this relationship. Furthermore, lifespace measurement could be applied to multiple affected population (e.g. diabetes, obesity, depression, physical disability including veterans’ injuries) to conduct both needs assessment and program evaluation of interventions that aim to address the impact of transportation, housing, and built environment policies on mobility in these populations. We recognize that mobile technology is evolving rapidly that may provide additional opportunities to measure neighborhood conditions. For example, wearable technology such as smart watches with GPS-enabled technology can track study participants as they move through their environment or are tagged by geolocated environmental sensors.

We should not lose sight of the fact that many of these emerging technologies collect data about conditions at specific locations within a geographic area. The atomistic or individualistic fallacy occurs when one attempts to generalize an individual relationship to a higher level of the hierarchy (e.g., group effect). This can also occur with geographic data when relationships from a specific location (or from several locations) are erroneously assumed to hold at an aggregate level (e.g., census tract). Therefore, the application of multilevel and spatial statistics to analyze these data are recommended to account for these hierarchies and for statistical inference among geographic areas [92]. Another approach is to use a GIS to approximate aggregate measures using the distance-area-for-clinical-care method which averages values from a finite number of locations that are proximal to a fixed location [93–95], Regardless of the method chosen, it should be noted that statistical models that incorporate the spatial (geographic) aspect of data collection should be employed to maximize the information contained in geographically referenced data.

Ethical considerations must also be taken into account when integrating spatial data into public health research and action. Geographically identifiable data is beneficial for helping public health research and practitioners to understand individuals as they move through their environment. However, this requires an examination of the ethics of using data in this manner, especially given individuals may be unaware that data collected about them in public spaces if being used for research purposes to which they have not consented. Clearly, the data collected by the emerging technologies that utilize publicly available images and posts and were not intended by the user to be categorized and analyzed in this manner. Sensitivity and caution should be exercised in this context when reporting analyses of these data, particularly due to the limited details in user agreements.

Finally, there seems to be tension between epidemiological studies that aim to collect data from well-defined populations using probabilistic sampling frames and data collected by emerging technologies that are unlikely representative of the general population. Weighting of the data collected by the latter method is recommended to obtain more representative samples. However, in some instances characteristics of the users are unknown, making weighting more difficult. Challenges also exist incorporating these data into existing methods, particularly when the data, obtained from emerging technologies, are not implemented by and not under the control of research teams.

## Conclusion

To most effectively and precisely measure built and social environment constructs, researchers should consider integrating new technology to assess these characteristics. Emerging technologies such as omnidirectional imagery, social media, drones, webcams, and crowdsourcing abound with data and may serve as effective and inexpensive tools to measure the ever-changing environment. By harnessing these technologies, public health research can not only monitor populations and the environment, but intervene using novel strategies to improve the public health.

## Abbreviations

AMOS: Archive of Many Outdoor Scenes
GIS: geographic information system
UAV: unmanned aerial vehicle
UAV: unmanned aircraft system
